# *α*-Ketoglutarate Improves Meiotic Maturation of Porcine Oocytes and Promotes the Development of PA Embryos, Potentially by Reducing Oxidative Stress through the Nrf2 Pathway

**DOI:** 10.1155/2022/7113793

**Published:** 2022-02-21

**Authors:** Qiang Chen, Leilei Gao, Jiannan Li, Yitian Yuan, Ruibin Wang, Yiqi Tian, Anmin Lei

**Affiliations:** Shaanxi Stem Cell Engineering and Technology Research Center, College of Veterinary Medicine, Northwest A&F University, Yangling 712100, China

## Abstract

*α*-Ketoglutarate (*α*-KG) is a metabolite in the tricarboxylic acid cycle. It has a strong antioxidant function and can effectively prevent oxidative damage. Previous studies have shown that *α*-KG exists in porcine follicles, and its content gradually increases as the follicles grow and mature. However, the potential mechanism of supplementation of *α*-KG on porcine oocytes during in vitro maturation (IVM) has not yet been reported. The purpose of this study was to explore the effect of *α*-KG on the early embryonic development of pigs and the mechanisms underlying these effects. We found that *α*-KG can enhance the development of early pig embryos. Adding 20 *μ*M *α*-KG to the in vitro culture medium significantly increased the rate of blastocyst formation and the total cell number. Compared with to that of the control group, apoptosis in blastocysts of the supplement group was significantly reduced. *α*-KG reduced the production of reactive oxygen species and glutathione levels in cells. *α*-KG not only improved the activity of mitochondria but also inhibited the occurrence of apoptosis. After supplementation with *α*-KG, pig embryo pluripotency-related genes (OCT4, NANOG, and SOX2) and antiapoptotic genes (Bcl2) were upregulated. In terms of mechanism, *α*-KG activates the Nrf2/ARE signaling pathway to regulate the expression of antioxidant-related targets, thus combating oxidative stress during the in vitro culture of oocytes. Activated Nrf2 promotes the transcription of Bcl2 genes and inhibits cell apoptosis. These results indicate that *α*-KG supplements have a beneficial effect on IVM by regulating oxidative stress during the IVM of porcine oocytes and can be used as a potential antioxidant for IVM of porcine oocytes.

## 1. Introduction

The maturation level of oocytes cultured *in vitro* and the ability of embryos to develop after fertilization are lower than those of oocytes cultured *in vivo*, and developmental retardation often occurs. This retardation may occur because oocytes cultured *in vitro* are sensitive to changes in the external environment, and there are differences between the *in vitro* culture environment and the internal environment. These differences may include the reactive oxygen species produced by the internal and external environments, the composition of the culture fluid, the O_2_ concentration, light, temperature, and pH value [1]. Porcine embryos produced *in vitro* have poor developmental potential, resulting in lower blastocyst yield and total cell numbers when compared with their *in vivo* counterparts [2–4]. The culture conditions are the main factors that affect blastocyst quality, whereas the intrinsic quality of the oocyte used for IVF has a great impact on the blastocyst yield [5]. In the process of *in vitro* maturation (IVM), reactive oxygen species (ROS) can be found in oocytes. The oxygen comes from the external medium and internal mitochondrial metabolism [6]. Regardless of the source, ROS can cause DNA damage, cell dysfunction, and apoptosis [7]. Previous studies have shown that vitamin C (VC) [8], astaxanthin [9], melatonin (MLT) [10], and caffeine [11] can improve oocyte quality and thus improve the results of assisted reproduction. Therefore, we investigated the mechanisms of antioxidative damage and explored the use of effective antioxidants in the culture medium, as an effective way to improve the development of pig embryos *in vitro* [12].

*α*-Ketoglutarate (*α*-KG) is an intermediate in the tricarboxylic acid cycle and a precursor of glutamine, which is widely present in organisms. *α*-KG is an effective free radical scavenger and broad-spectrum antioxidant. The enzyme and its metabolites directly scavenge ROS and reduce oxidative damage to cells and their components, including the mitochondria [13, 14]. However, the way in which *α*-KG inhibits oxidative stress in oocytes has not been studied. Therefore, we adopted a porcine mammalian system that is very similar to human germ cells in many respects and investigated the hypothesis that *α*-KG can scavenge free radicals generated during oxidative stress in oocytes, maintaining the quality of oocytes.

In this study, we explored the effects of *α*-KG supplementation during *in* vitro maturation (IVM) on the maturation of porcine oocytes and the subsequent development of parthenogenetic embryos. We analyzed the effects of *α*-KG on ROS levels, apoptosis, glutathione content, mitochondrial distribution, and oxidative stress-related gene expression in pig oocytes. We found that *α*-KG could activate the Nrf2/ARE signaling pathway to regulate the expression of downstream antioxidant-related target genes to enhance the antioxidant capacity of pig oocytes. These results help to clarify the mechanism of the beneficial effects of *α*-KG on oocyte quality and subsequent parthenogenetic embryo development.

## 2. Materials and Methods

FITC/Cy3-labeled goat anti rabbit/mouse IgG antibody was purchased from the Beyotime Biotechnology (Beijing, China). Nrf2, Caspase3, and Bcl2 antibodies were purchased from Wanlei Biotechnology Co., Ltd. (Shenyang, China). Unless otherwise specified, all the reagents were purchased from Sigma-Aldrich (Shaanxi, China).

### 2.1. In Vitro Maturation (IVM) and *α*-KG Treatment

OOvaries were collected at a local abattoir and transported to the lab within 2 h in a thermos filled with physiological saline at 30–37°C. COCs from follicles 3–6 mm in diameter were aspirated using a 12-gauge needle attached to a 10 mL disposable syringe. The obtained follicular fluid is timely injected into a 15 mL glass centrifuge tube placed in a thermostat water bath with 37°C. After standing for 10-15 minutes, obvious precipitation can be observed at the bottom of the centrifuge tube. Discard the supernatant carefully with a sterile pasteurized tube and leave 2 mL. Then, mix it with TCM-199 (12340, GIBCO, Shanxi, China), and move it to a 90 mm sterile culture dish for oocyte collection. Under the stereomicroscope, COCs with uniform cytoplasm and three or more layers of cumulus granulosa cells were selected for the experiment. The in vitro maturation medium was composed of TCM-199 (11150, GIBCO), 0.1% polyvinyl alcohol, 3.05 mm D-glucose, 0.91 mm sodium pyruvate, 0.57 mm cysteine, 75 *μ*g/mL penicillin (Harbin sixth pharmaceutical factory), 50 *μ*g/mL streptomycin (North China Pharmaceutical Co., Ltd.), and 10 *μ*g/mL streptomycin, 10 IU/mL PMSG (Sansheng Pharmaceutical Co., Ltd.) and 10 IU/mL hCG (Sansheng Pharmaceutical Co., Ltd.), 10 ng/mL EGF, and 2.5 IU/mL FSH (Sansheng Pharmaceutical Co., Ltd.). After washing with the balanced IVM medium three times, the medium was evenly distributed into a four-well plate containing 500 *μ*L mature medium and cultured in a 5% CO_2_ incubator at 38.5°C for 42-44 h. Different concentrations of *α*-ketoglutarate (0 *μ*M, 10 *μ*M, 20 *μ*M, 50 *μ*M, and 100 *μ*M) were added to 500 *μ*L mature medium per well.

### 2.2. Parthenogenetic Activation and Embryo Culture In Vitro

Oocytes with a polar body will be collected for parthenogenetic activation. Oocytes were placed in an activation chamber with electrodes 1 mm apart containing activating medium. The distance between each oocyte was about 2 mm, and they could not adhere to the wall. Then, the electrofusion instrument was started to activate. Activation was induced on a BTX Electro-Cell Manipulator (BTX ECM2100, San Diego, CA, United States). Parameters of electrofusion instrument are as follows: mode—LV, pulses—02, voltage—90 V, interval—0.10 s, p.length—10 *μ*s, and polyarty—unipolar. After electrical activation, the activated oocytes were then washed with the chemical activation solution (6-DAMP + CB) and then activated for about 2 hours. Finally, they were transferred to PZM-3 medium and cultured under 5% CO2 in air at 38.5°C. The cleavage rate was observed 48 hours after activation, and blastocyst rate was observed 144 hours after activation.

### 2.3. Immunofluorescence Staining

Denuded MII oocytes or blastocysts were collected and fixed in 4% paraformaldehyde (PFA) in PBS overnight at 4°C. After being washed 3 times in PBS, 5 min each time, oocytes were permeabilized in 0.2% TritonX-100 (in PBS) for 30 min at room temperature. Subsequently, the samples were washed three times with PBS and then transferred into the immunofluorescence staining blocking solution. After incubation at room temperature for 3-4 h, the samples were directly transferred into the primary antibody overnight at 4°C. On the second day, the oocytes or blastocysts were transferred into PBS from the first antibody, washed three times, and incubated with secondary antibody for 2 h in the dark at room temperature. After washing three more times, Hoechst 33342 (10 *μ*g/mL) was used to stain nuclei for 5-10 min at room temperature. Samples were mounted on glass slides and examined with a Fluorescence phase contrast inverted microscope (EVOS, Thermo Fisher Scientific).

### 2.4. Detection of Reactive Oxygen Species (ROS) and L-Glutathione (GSH)

A DCFH diacetate (DCFH-DA) kit (Beyotime Biotechnology, S0033, China) and CellTracker™ fluorescent probes (Thermo Fisher Scientific, C12881, China) were used to examine the level of intracellular ROS and GSH levels during oocyte maturation. The oocytes were incubated in PBS-PVA containing 10 *μ*M 2′,7′-DCFH or 10 *μ*M 4-CMF2HC for 15 min. After washing the oocytes three times in PBS-PVA, images were captured using a fluorescence microscope (Nikon, Tokyo, Japan).

### 2.5. Mitochondrial Membrane Potential (*ΔΨ*m) Detection

MitoProbe JC-1 Assay kits (M34152, Thermo Fisher Scientific, Waltham, MA, United States) were used to detect mitochondrial membrane potential (*ΔΨ*m). The steps of JC-1 staining are the same as above, and 2 *μ*M JC-1 was added in TCM-199 culture medium. The change in JC-1 from red (∼590 nm) to green (∼529 nm) fluorescence was used to detect a decrease in mitochondrial membrane potential. The oocytes were washed three times with preheated PBS, then transferred into JC-1 staining working solution, and incubated in incubator for 20 min. The oocytes were washed three times with pre cooled staining buffer and then photographed as soon as possible with a fluorescence microscope (Nikon, Tokyo, Japan). The ratio of red to green fluorescence intensity was analyzed using ImageJ software.

### 2.6. Determination of Mitochondria Distribution

To assess mitochondria distribution, after washing oocytes with prebalanced IVM medium for 3 times, oocytes were transferred into 250 nM MitoTracker Green staining solution, incubated in incubator for 30 min, and then washed with IVM medium for three times. Finally, oocytes were transferred into droplets and immediately photographed with a fluorescence microscope (Nikon, Tokyo, Japan).

### 2.7. RNA Extraction and Quantitative Reverse Transcription PCR (RT-qPCR)

A total of 10 oocytes were harvested for RNA extraction using acidic Tyrode's solution (pH 2.5) and lysates (DTT 5 mmol/L, RNAse 20 U/mL, NP-40 1%, in Nuclease-free Water) [15]. Briefly, the selected oocytes were placed in acidic Tyrode's solution for 20–60 s to remove the zona pellucida, then transferred to lysates (10 *μ*L) and placed in a −80°C freezer for at least 15 min. Next, cDNAs were obtained by SuperScript® III One-Step RT–PCR System with Platinum® Taq high fidelity DNA polymerase (Invitrogen, Beijing, China) to detect the expression of genes, according to the AceQ qPCR SYBR® Green Master Mix (Vazyme Biotech, Nanjing, China) by qRT-PCR. Briefly, 2 *μ*L of forward and reverse primers mix (S-table 2), 5 *μ*L of SYBR Green mix, and 1 *μ*L of nuclease-free water were mixed and distributed into hardshell 96-well skirted PCR plates (Bio-Rad Laboratories) before adding 2 *μ*L of diluted cDNA (150 ng/*μ*L). Using CFX96 instrument (Bio-Rad Laboratories), the qPCR was performed under the following conditions: 95°C 5 min; 95°C 10 s, 60°C 30 s, Reps 40; 95°C 15 s, 60°C 60 s, 95°C 15 s. Each cDNA sample was applied in duplicate (three biological replicates). The mRNA abundance of genes was estimated using GAPDH as a reference gene where the transcription level of each gene in the untreated control was set as 1.

### 2.8. Detection of Early Oocyte Apoptosis

Annexin V staining kit (Vazyme, Nanjing, China) was used to detect early apoptosis level in oocytes. Prepare the dye working solution according to the instruction. The oocytes were washed three times with the preheat PBS; then, after washing with binding buffer, the oocytes were incubated in the staining working solution for 15 minutes in the dark at room temperature. Then, oocytes were washed with PBS for three times, and the fluorescent microscope (Nikon, Tokyo, Japan) could be used for observation and photo taking.

### 2.9. Staining of the Total Cell Number of Blastocysts

Fresh blastocysts were fixed in 4% paraformaldehyde at 4°C overnight. The next day, the cells were washed with PBS for three times and then permeated with 0.2% Triton X-100. After being incubated at room temperature for 30 min, the oocytes were washed with PBS for three times and transferred to DAPI for nucleus staining. The oocytes were incubated in the dark for 5-10 min at room temperature and then washed with PBS for three times. The total number of cells was observed and photographed with a fluorescent microscope (Nikon, Tokyo, Japan).

### 2.10. Statistical Analysis

The oocyte and embryo experiments were repeated at least three times. ImageJ was used for fluorescence intensity statistics, and GraphPad 8.0 program was used to analyze and plot all data. The results were expressed as the mean ± standard error and passed Tukey's test. *P* < 0.05 was considered statistically significant (^∗^*P* < 0.05,  ^∗∗^*P* < 0.01, and^∗∗∗^*P* < 0.001 showed different significances, respectively).

## 3. Results

### 3.1. Effects of *α*-KG Supplementation during the IVM Process on Porcine Oocyte Maturation

**Figure 1:**
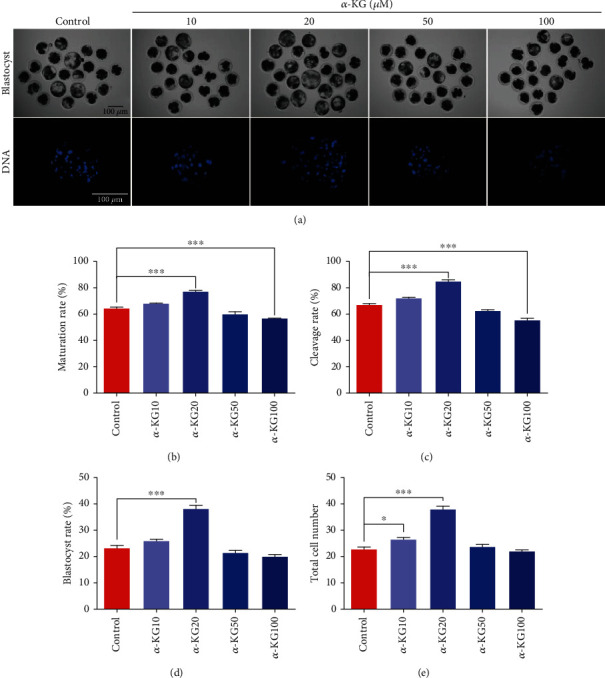
Effects of *α*-ketoglutarate on the developmental competence of porcine PA embryos. (a) Representative images of embryo development (top row) and Hoechst 33,342 staining of blastocysts on day 7 (bottom row) in the control and *α*-ketoglutarate groups. Scale bar = 100 *μ*m. (b) Effects of *α*-ketoglutarate on PA embryonic development. (c) Effects of *α*-ketoglutarate on blastocyst rate. (d) Effects of *α*-ketoglutarate on the cell numbers of blastocysts (mean ± SEM of 1938 oocytes). Significant differences are represented with ^∗^*P* < 0.05 and ^∗∗∗^*P* < 0.001. The data were obtained from three separate experiments.

When evaluating the effect of *α*-KG during the process of pig oocyte maturation, cumulus-oocyte complexes (COCs) were cultured with various concentrations of *α*-ketoglutarate (10, 20, 50, and 100 *μ*M), and the polar body exclusion (PBE) rate of porcine oocytes was evaluated at the end of IVM. We observed that high concentrations of *α*-KG could reduce the PBE rate, while 100 *μ*M *α*-KG could significantly decrease the PBE rate of oocytes (S-table 1), and 10 and 20 *μ*M *α*-KG could significantly increase the PBE rate of oocytes (S-table 1), and the difference in the 20 *μ*M group was extremely significant (Con vs. 20 *μ*M: 64.7% ± 1.20% vs. 76.83% ± 1.05%, *P* < 0.001) (Figure 1(b)).

### 3.2. Effect of *α*-KG on the Developmental Potential of Pig Oocytes *In Vitro*

We found that supplementation with 20 *μ*M *α*-ketoglutarate during the IVM process not only significantly increased the rate of two-cell and blastocyst formation of the PA embryos (Figures 1(a), 1(c), and 1(d); 23.07% ± 1.16% vs. 38.07% ± 1.34% on day 7; *P* < 0.01) but also increased the total cell number (Figure 1(e); 22.67% ± 0.88% vs. 37.67% ± 1.45% on day 7; *P* < 0.01) of blastocysts compared with that in the control groups. These results showed that *α*-KG supplementation during the IVM process not only promoted the developmental competence of porcine early embryos derived from PA but also improved their quality.

### 3.3. Effects of *α*-KG Supplementation during the IVM Process on Oxidative Stress Resistance of Porcine Oocytes

**Figure 2:**
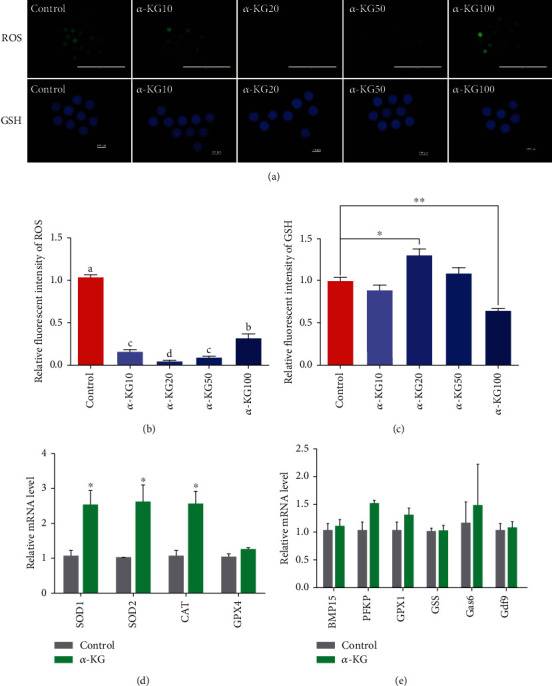
Effects of *α*-KG on the formation of ROS and GSH in mature oocytes. (a) Representative pictures of reactive oxygen species and glutathione staining. (b) Analysis of relative fluorescence intensity of ROS in the control group and the *α*-KG-supplemented group. (c) Analysis of relative fluorescence intensity of GSH in the control group and the *α*-KG-supplemented group. (A, B) *P* < 0.05, ^∗^*P* < 0.05, and ^∗∗^*P* < 0.01; SEM: standard error of mean. (d) The effect of adding *α*-KG on the mRNA expression of antioxidant-related genes in mature oocytes. ^∗^*P* < 0.05; SEM: standard error of mean. (e) Relative mRNA expression of oocyte maturation-related genes (BMP15, PFKP, GPX1, GSS, Gas6, and Gdf9) in the control group and the *α*-KG addition group; ^∗^*P* < 0.05; SEM: standard error of mean.

Mitochondrial dysfunction during the *in vitro* maturation of oocytes usually induces oxidative stress. We tested the levels of GSH and ROS in pig oocytes after supplementation with *α*-KG.  Figure 2 shows that the overall gradient trend of the *α*-KG addition group was significantly lower than that of the control group, and the 20 *μ*M *α*-KG group had the lowest ROS level (*P* < 0.05) (Figures 2(a) and 2(b)). This result is consistent with the maturation and development rate of oocytes above. When *α*-KG was added to the IVM medium, the GSH level of the 20 *μ*M *α*-KG group was higher than that of the control group (*P* < 0.05) (Figures 2(a) and 2(c)). The above results show that *α*-KG could reduce the levels of ROS and increase the GSH levels, increasing the antioxidant capacity of the oocytes and thereby improving their quality. Since the 20 *μ*M group showed the most significant improvement, we chose the 20 *μ*M *α*-KG supplement group for testing in subsequent experiments.

After demonstrating that *α*-KG could increase the antioxidant capacity of oocytes by reducing the content of ROS and increasing the level of GSH, we examined the role of the antioxidant-related genes *SOD1*, *SOD2*, *CAT*, and *GPX4* and oocyte maturation. We measured the mRNA levels of the genes *BMP15*, *PFKP*, *GPX1*, *GSS*, *Gas6*, and *Gdf9*. The mRNA levels of *SOD1*, *SOD2*, and *CAT* in oocytes of the *α*-KG group were significantly higher than those of the control group (*P* < 0.05, Figure 2(d)), and there was no significant difference in the mRNA level of the *GPX4* gene. The mRNAs of the cytoplasmic maturation genes *BMP15*, *PFKP*, *GPX1*, *GSS*, *Gas6*, and *Gdf9* were not significantly different from those of the control group (Figure 2(e)), indicating that *α*-KG enhanced the expression of oocyte antioxidant genes and cytoplasmic maturation genes. In this way, the oxidative stress caused by *in vitro* culture can be relieved and the quality of oocytes can be improved.

### 3.4. Effects of *α*-KG Supplementation during the IVM Process on Porcine Oocyte Mitochondrial Function

**Figure 3:**
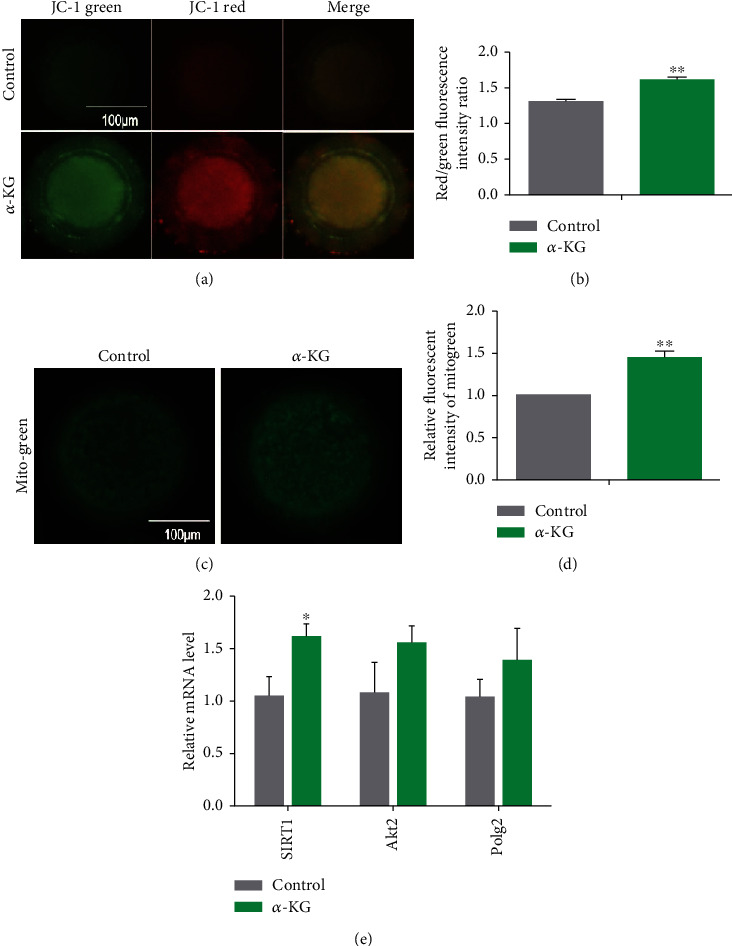
The effect of *α*-KG on the mitochondrial membrane potential of mature oocytes. (a) JC-1 staining representative pictures of the control group and the *α*-KG addition group. (b) The relative fluorescence intensity analysis of the JC-1 red/green light in the control group and the *α*-KG addition group. ^∗∗^*P* < 0.01; scale bar = 50 *μ*m; SEM: standard error of mean. (c) MitoTracker-Green staining representative pictures of the control group and *α*-KG-supplemented group. (d) The relative fluorescence intensity analysis of MitoTracker-Green of the control group and *α*-KG-supplemented group; ^∗∗^*P* < 0.01; scale bar = 50 *μ*m; SEM: standard error of mean. (e) Relative mRNA expression of mitochondrial-related genes (*SIRT1*, *Akt2*, *Polg2*) in the control group and the *α*-KG addition group.

Under normal physiological conditions, the intracellular ROS content is balanced. Excessive levels of ROS are not conducive to mitochondrial activity and cause DNA damage. There are many mitochondria in oocytes, and their activity is important for the quality of the oocytes. Normal mitochondrial membrane potential (MMP) is conducive to the oxidative phosphorylation of oocytes. Therefore, we used the JC-1 detection kit to detect the mitochondrial membrane potential of mature oocytes in the control group and the *α*-KG-supplemented group. The ratio of JC-1 red light/green light in the *α*-KG addition group was higher than that in the control group, and the difference was extremely significant (1.3% ± 0.03% vs. 1.61% ± 0.05%, *P* < 0.01, Figures 3(a) and 3(b)), indicating that *α*-KG can increase the MMP of pig oocytes.

We used MitoTracker-Green fluorescent probe to detect the distribution of mitochondria in mature oocytes of the two groups. The higher the developmental potential of the oocytes, the stronger the activity of the mitochondria, and the more uniform the distribution. It was found that the mitochondrial positioning of the *α*-KG addition group was more uniform, and the fluorescence intensity of the mitochondria was stronger (1.07% ± 0.07% vs. 1.45% ± 0.07%, *P* < 0.01, Figures 3(c) and 3(d)). This observation indicates that *α*-KG increases the mitochondrial content during oocyte maturation and the distribution of the mitochondria is more uniform. We investigated the mRNA expression of the mitochondria-related genes *SIRT1*, *Akt2*, and *Polg2*. The mRNA level of *SIRT1* in oocytes to which *α*-KG was added was significantly higher than that in the control group (*P* < 0.05, Figure 3(e)). Therefore, these results show that *α*-KG improved the mitochondrial function of pig oocytes.

### 3.5. Effects of *α*-KG Supplementation during the IVM Process on Apoptosis in MII Oocytes

**Figure 4:**
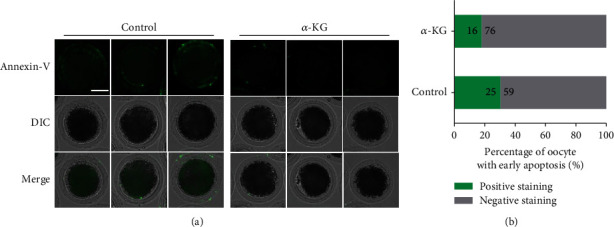
The effect of *α*-KG on early apoptosis of mature oocytes. (a) Representative staining pictures of Annexin V-positive results in the control group and Annexin V-negative results in the *α*-KG addition group. (b) The percentage of oocytes with positive Annexin V results in the control group and the *α*-KG addition group; scale bar = 50 *μ*m.

Because excessive ROS levels can induce apoptosis, we used Annexin V-FITC fluorescent probe to detect early apoptosis level of mature pig oocytes in the two groups. In accordance with previous reports [16, 17], a green circular fluorescent signal observed on the outside of the cell membrane of the oocyte was defined as a positive result for Annexin V, and lack of the signal was a negative result for Annexin V (Figure 4(a)). We analyzed the oocytes of the two groups individually. In the control group, 29.76% (25/84) of the oocytes were Annexin V positive, but in the *α*-KG addition group, only 17.39% (16/92) of oocytes were positive for Annexin V (Figure 4(b)). This result indicates that *α*-KG reduced the occurrence of apoptosis in early oocytes.

**Figure 5:**
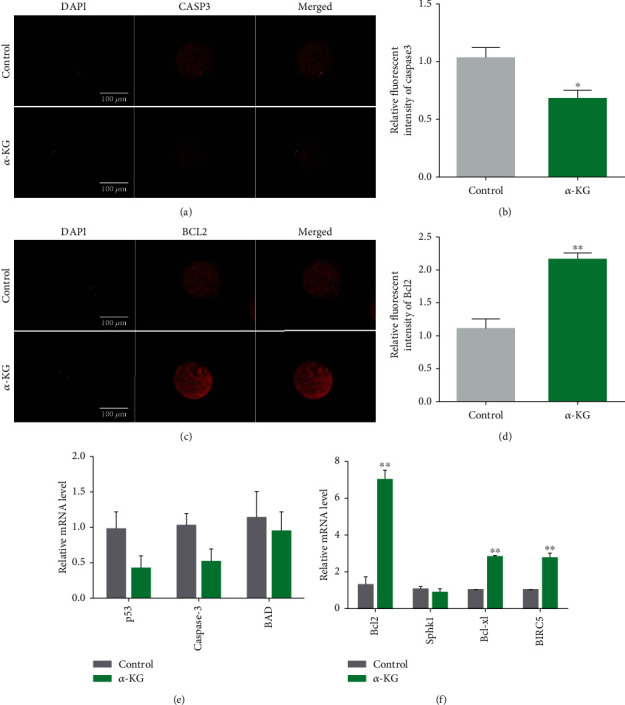
Effects of *α*-KG on Caspase3 and Bcl2 of mature oocytes. (a) Representative pictures of Caspase3 immunofluorescence staining in the control group and *α*-KG-supplemented group. (b) Analysis of the relative fluorescence intensity of Caspase3 in the control group and *α*-KG-supplemented group. (c) Representative pictures of Caspase3 immunofluorescence staining of blastocysts in each group. (d) Analysis of Bcl2 relative fluorescence intensity of blastocysts in each group. (e) The relative expression of proapoptotic genes (*p53*, *Caspase3*, and *BAD*) mRNA in the control group and the *α*-KG addition group. (f) The relative expression of mRNA of antiapoptotic genes (*Bcl2*, *Sphk1*, *Bcl-xl*, and *BIRC5*) in the control group and the *α*-KG addition group. ^∗^*P* < 0.05 and ^∗∗^*P* < 0.01; SEM: standard error of mean.

We measured the expression of proapoptotic genes and antiapoptotic genes in the two groups of mature oocytes using immunofluorescence staining. As shown in Figure 5, the fluorescence intensity of the proapoptotic gene Caspase3 in the *α*-KG addition group was significantly weaker than that of the control group (1.15% ± 0.15% vs. 0.44% ± 0.06%, *P* < 0.01, Figures 5(a) and 5(b)). The fluorescence intensity of the antiapoptotic gene Bcl2 in the *α*-KG addition group was significantly weaker than that of the control group (1.11% ± 0.14% vs. 2.17% ± 0.08%, *P* < 0.01, Figures 5(c) and 5(d)). These experimental results showed that adding *α*-KG to the maturation medium could effectively enhance the antiapoptotic ability of oocytes and delay the occurrence of apoptosis.

The mRNA expression of genes associated with apoptosis in mature oocytes was evaluated, as shown in Figures 5(e) and 5(f). The mRNA levels of BAD, Caspase3, and P53 were decreased in oocytes treated with *α*-KG compared with the control group, but the difference was not significant. After *α*-KG treatment, the mRNA expression of antiapoptotic genes was increased in oocytes treated with *α*-ketoglutarate compared with the control group (*P* < 0.01). These results indicate a protective role of *α*-KG against oocyte apoptosis.

### 3.6. Effect of *α*-KG on PA Embryo Pluripotency

**Figure 6:**
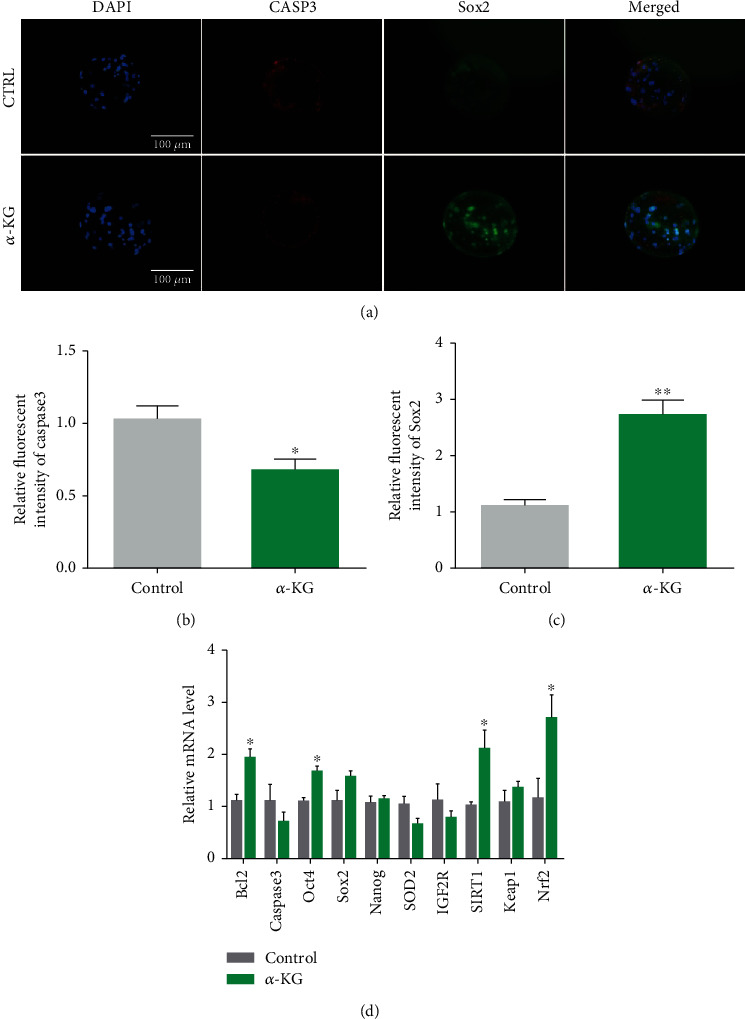
Effects of *α*-KG on gene expression in parthenogenetic embryos. (a). Representative pictures of Sox2 and Caspase3 immunofluorescence costaining in the control group and the *α*-KG-supplemented group. (b) Representative pictures of Caspase3 immunofluorescence staining of blastocysts in each group. (c) Analysis of Sox2 relative fluorescence intensity of blastocysts in each group. (d) Effects of *α*-KG on mRNA expression of developmental potential-related genes in parthenogenetic embryos. ^∗^*P* < 0.05 and^∗∗^*P* < 0.01; scale bar = 100 *μ*m. SEM: standard error of mean. ^∗^*P* < 0.05.

We performed immunofluorescence analysis on the blastocysts in the control group and the *α*-KG group and costained with Sox2-FITC and Caspase3-CY3. The *Sox2* fluorescence intensity of blastocysts in the 20 *μ*M group was significantly higher than that of the control group (1.13% ± 0.09% vs. 2.74% ± 0.25%, *P* < 0.01, Figures 6(a) and 6(c)), and the *Caspase3* fluorescence intensity of blastocysts in the 20 *μ*M group was significantly higher than that of the control group (1.03% ± 0.08% vs. 0.68% ± 0.07%, *P* < 0.05, Figures 6(a) and 6(b)). These results indicate that *α*-KG could enhance the expression of *Sox2* in the subsequent parthenogenetic embryos of mature oocytes and reduce the expression of Caspase3.

The mRNA levels of genes related to the developmental potential of parthenogenetic blastocysts*—Bcl2*, *Caspase3*, *Oct4*, *Nanog*, *SOD2*, and *IGF2R*—were measured. Compared with the control group, the mRNA expression of the antiapoptotic gene *Bcl2* of the blastocysts in the *α*-KG addition group was significantly higher than that of the control group (*P* < 0.05, Figure 6(d)). The mRNA expression of the pluripotency genes *Oct4* and *Sox2* also increased significantly (*P* < 0.05, Figure 6(d)). We measured the mRNA expression of *Nrf2* and *Keap1* in the blastocysts in the control group and the *α*-KG group. The mRNA expression of *Nrf2* in parthenogenetic activated embryos obtained from mature oocytes in the *α*-KG addition group was significantly higher than that in the control group (1.1% ± 0.04% vs. 2.02% ± 0.67%, *P* < 0.05, Figure 6(d)).

### 3.7. Inverse Effects of *α*-KG and Brusatol Treatment during IVM

**Figure 7:**
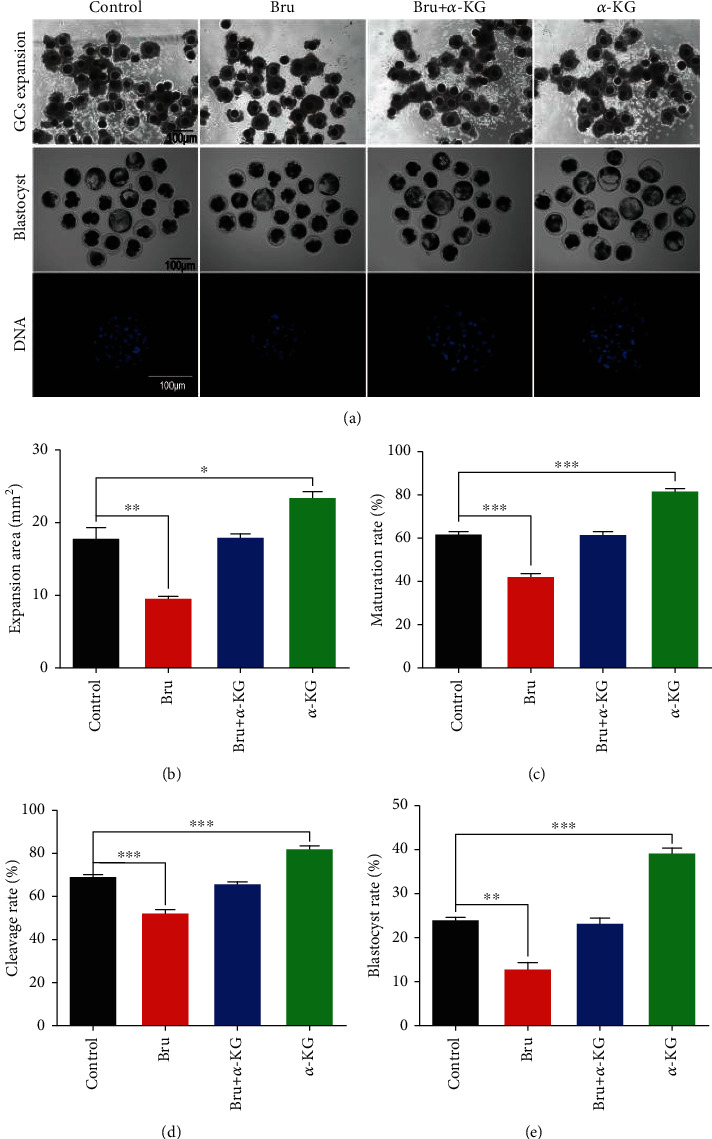
Evaluation of treatments with *α*-KG and brusatol on porcine COCs and their competences in PA embryo development. (a) The effects of treatment with 20 *μ*M *α*-KG and 12 nM brusatol on CC expansion and subsequent embryonic development were evaluated. The four experimental groups (control group, brusatol group, *α*-KG group, and brusatol+*α*-KG group), COCs cumulus granulosa cell expansion bright field image, parthenogenetic activated embryo blastocyst bright field image, and Hoechst 33342 nuclear staining image. (b) Expansion of cumulus granulosa cells in each group of COCs. (c) Maturity rate of each group. (d) Cleavage rate of parthenogenetic embryos in each group. (e) Blastocyst rate of parthenogenetic embryos in each group. ^∗^*P* < 0.05,  ^∗∗^*P* < 0.01, and^∗∗∗^*P* < 0.001; scale bar = 100 *μ*m. SEM: standard error of mean.

In order to further confirm that *α*-KG and brusatol have opposite functions when regulating Nrf2, they were used together during IVM, and their effects on subsequent PA embryonic development were evaluated. According to previous literature reports [18], an appropriate working concentration of brusatol is 12 nM. The collected GV stage pig oocytes were divided equally into four groups: the control, brusatol, *α*-KG, and cotreatment with *α*-KG and brusatol (cotreatment) (Figure 7(a)). After culture, we evaluated the cumulus expansion area of each experimental group. The *α*-KG-treated group showed the highest cumulus expansion area, as shown in Figure 7(b) (17.87% ± 1.49% vs. 9.57% ± 0.34%, *P* < 0.01; 17.87% ± 1.49% vs. 23.53% ± 0.87%, *P* < 0.05, respectively). All experimental groups showed significant differences in the cumulus expansion area. We analyzed the discharge rate of the first polar body. The *α*-KG treatment group showed a higher maturity rate than the control group, the brusatol group, and the combination treatment group (79.43% vs. 62.36% and 39.71% vs. 58.35%, respectively, *P* < 0.001) (Figure 7(c)), and the brusatol treatment group had the lowest maturity rate. During the development of parthenogenetic embryos, the cleavage rate of the control group and the *α*-KG treatment group was higher than that of the brusatol group and the combination treatment group (70.16% and 81.44% vs. 51.96% and 64.25%, respectively, *P* < 0.001) (Figure 7(d)). The *α*-KG treatment group showed a higher blastocyst formation rate than the control group, the brusatol group, and the combination treatment group (38.36% vs. 24.71% and 12.35% vs. 22.43%, respectively, *P* < 0.05) (Figure 7(e)), while the brusatol treatment group had the lowest blastocyst formation rate.

**Figure 8:**
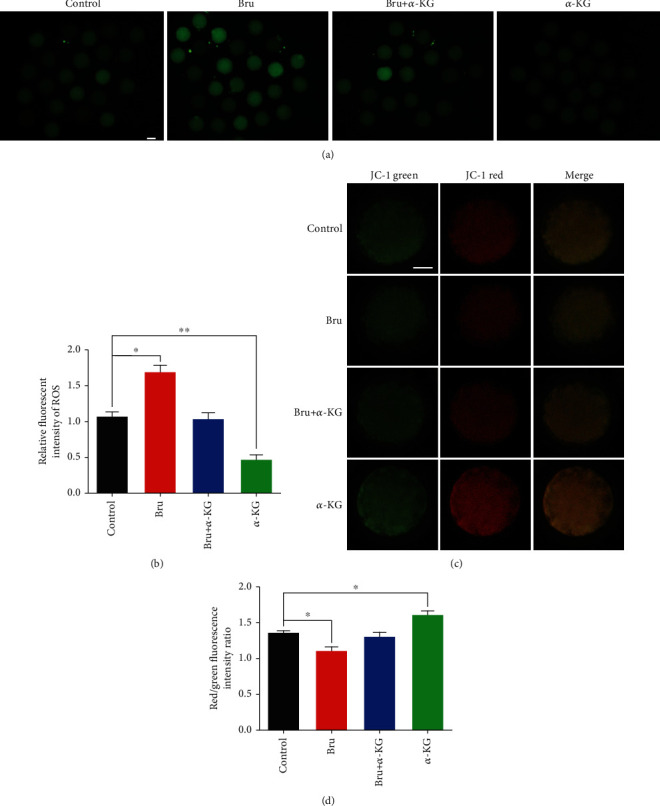
The effect of cotreatment with brusatol and *α*-KG on ROS and JC-1 of oocytes. (a) Representative pictures of ROS staining in each group (control group, brusatol group, *α*-KG group, and brusatol+*α*-KG group). (b) ROS relative fluorescence intensity analysis of each group. (c) Representative pictures of JC-1 staining in each group. (d) JC-1 red light/green light relative fluorescence intensity analysis. ^∗^*P* < 0.05 and^∗∗^*P* < 0.01; scale bar = 50 *μ*m. SEM: standard error of mean.

All treatment groups were tested for ROS and JC-1 (Figures 8(a) and 8(c)). ROS levels were the lowest in the *α*-KG-treated group and highest in the brusatol-treated group (*P* < 0.05 and *P* < 0.01) (Figure 8(b)). The cotreated group showed restored ROS levels when compared to the brusatol-treated group. The MMP was the highest in the *α*-KG-treated group and the lowest in the brusatol-treated group (*P* < 0.05) (Figure 8(d)). Compared with the brusatol-treated group, the cotreated group showed restored MMP. In summary, we partially verified that *α*-KG positively affected oocyte maturation and embryo development, in keeping with the results of previous studies.

**Figure 9:**
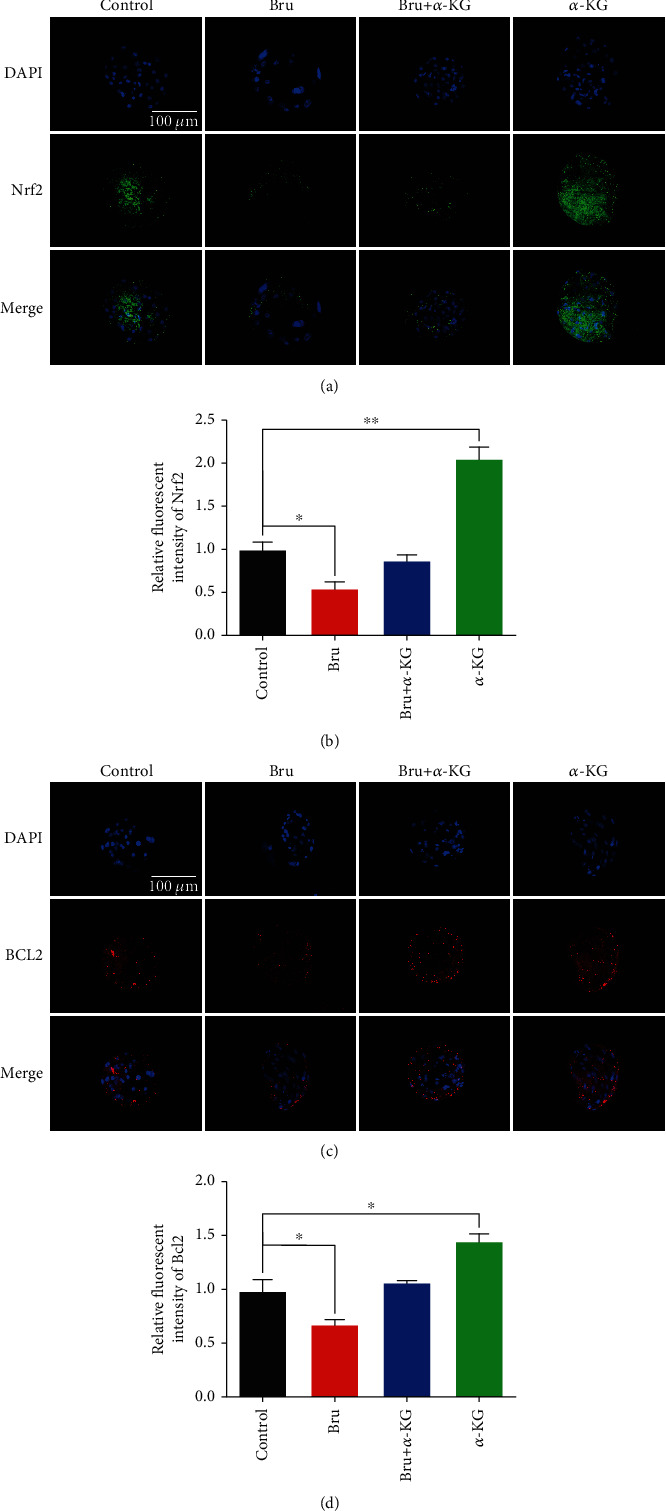
Protein analysis of protein of interest in porcine blastocysts by immunocytochemistry. (a) Representative pictures of Nrf2 immunofluorescence staining of blastocysts in each group (control group, brusatol group, *α*-KG group, and brusatol+*α*-KG group). (b) Analysis of the relative fluorescence intensity of Nrf2 in each group of blastocysts. (c) Representative pictures of Nrf2 immunofluorescence staining of blastocysts in each group. (d) Analysis of Bcl2 relative fluorescence intensity of blastocysts in each group. ^∗^*P* < 0.05,  ^∗∗^*P* < 0.01, and^∗∗∗^*P* < 0.001; scale bar = 100 *μ*m. SEM: standard error of mean.

In order to clarify the role of *α*-KG and brusatol at the protein level, we performed immunofluorescence staining of blastocyst Nrf2 in the four groups, to demonstrate whether *α*-KG upregulated the expression of Nrf2. The Nrf2 fluorescence intensity was the strongest in the *α*-KG group (Con vs. *α*-KG: 0.98% ± 0.1% vs. 2.03% ± 0.15%, *P* < 0.01, Figures 9(a) and 9(b)), in agreement with the results of previous studies. The protein expression level of the antiapoptotic gene Bcl2 was also measured. Compared with the other groups, the fluorescence intensity of Bcl2 in the *α*-KG group was significantly increased (Con vs. *α*-KG: 0.97% ± 0.11% vs. 1.43% ± 0.08%, *P* < 0.05, Figures 9(c) and 9(d)) and showed no significant difference compared with the brusatol-treated group, indicating that *α*-KG could enhance the antiapoptotic ability of oocytes and reduce the level of apoptosis caused by brusatol.

## 4. Discussion

The quality of oocytes is a cornerstone of embryonic development, and the oxidative stress caused by *in vitro* culture is one of the key problems that oocytes cannot avoid. Based on previous studies, we found that many reports had investigated the addition of antioxidants such as mogroside, phloridin, or kaempferol to the *in vitro* culture process to combat oxidative stress, but the effects were not satisfactory [19–21]. It is currently recognized that the antioxidant effects of melatonin can improve the nuclear and qualitative maturation of oocytes. Subsequent studies have found that melatonin can relieve fat inflammation by increasing *α*-KG [22]. This observation is consistent with the results found by Liu Guoshi and others. During their research on the function and mechanism of melatonin in the field of reproduction, they also found that melatonin could increase the level of *α*-KG. However, few studies have investigated the effects of *α*-ketoglutarate supplementation during IVM on porcine oocyte maturation and the subsequent development of parthenogenetic embryos or the potential mechanism by which *α*-KG improves the quality of porcine oocytes. In this study, we found that 20 *μ*M *α*-ketoglutarate promoted porcine oocyte maturation and significantly enhanced the subsequent development and quality of parthenogenetic embryos *in vitro*. In order to elucidate the underlying mechanism, we studied the effects of *α*-KG on the levels of GSH and ROS in porcine oocytes, the occurrence of early cell apoptosis, the mitochondrial distribution, and the expression of gene involved in oxidative stress.

Under normal circumstances, cells maintain their ROS levels in a balanced state [23]. The *in vitro* culture environment produces high levels of ROS in oocytes than *in vivo*, without an accompanying increase in antioxidant capacity, leading to higher oxidative stress in oocytes [24]. In normal aerobic respiration, the redox intermediate step in the mitochondrial electron transport chain continuously produces ROS [25]. The formation of ROS also serves as a necessary intermediate in various enzymatic reactions. Common ROS species include O^2-^, -OH, and H_2_O_2_ [26]. Elevated ROS levels can produce cell membrane damage, DNA mutations and breaks, and mitochondrial dysfunction, which will eventually lead to meiosis arrest in oocytes and embryonic development [27, 28]. *α*-KG has an antioxidant function and plays an important role in the elimination of ROS in the organism [13]. It has been determined that *α*-KG can act as a true antioxidant. It can directly react with hydrogen peroxide to form succinate, water, and carbon dioxide. Subsequent studies have shown that *α*-KG can be used as a source of glutamic acid for the synthesis of glutathione. The concentration of GSH in pig oocytes matured *in vivo* is almost 4 times that of pig oocytes matured in vitro [29]. This difference may be caused by the difference in oxygen content inside and outside the body. The oxygen concentration in the maturation medium *in vitro* is almost three times that in the reproductive tract *in vivo*. Therefore, during the *in vitro* maturation process, the oocytes need to exhaust the intracellular GSH to resist the damage caused by oxidation, resulting in a decrease in GSH during maturation. The experimental results showed that the addition of *α*-KG during the culture process reduced the ROS levels of pig oocytes and increased the GSH levels. This observation indicates that *α*-KG enhances the overall antioxidant capacity of oocytes. This effect may occur because *α*-KG can promote cell metabolism and activate the antioxidant system in the cell by upregulating the biosynthesis of glutathione, thereby enhancing the resistance to oxidative stress.

Mitochondria are essential organelles in embryos, because they are a key component of the metabolic mechanism responsible for supplying the energy consumed during maturation [30] and are also the main generators of free radicals in mammals [31]. When the level of ROS in oocytes is too high, it has an adverse effect on mitochondria, inhibits mtDNA replication, and increases the risk of mtDNA mutations [32]. It also affects the MMP, which reduces the oxidative phosphorylation reaction and ATP synthesis [33], hinders the normal migration of mitochondria, causes poor mitochondrial aggregation, and ultimately promotes oocyte apoptosis and reduces the development ability of oocytes. Our experiments demonstrated that the mitochondrial activity increased in the *α*-KG-treated oocytes compared with control oocytes. The distribution of the mitochondria is less even in the control group than in the *α*-KG groups. Therefore, the result of our increased mitochondrial activity of oocytes may be related to the cytoprotective effect of *α*-KG and its inherent ROS scavenging characteristics, which not only reduces the level of ROS but also protects the function of mitochondria and increases the membrane potential and its distribution in the oocyte is more even.

To test whether the effect of *α*-KG during IVM is related to changes in gene expression, we analyzed the expression of candidate genes involved in oxidative stress, embryonic development, and embryo quality. We measured the mRNA expression of related genes, including the antioxidant-related genes *SOD1*, *SOD2*, *CAT*, and *GPX4*; the antiapoptosis-related genes *Bcl2*, *Sphk1*, *Bcl-xl*, and *BIRC5*; the proapoptosis-related genes *p53*, *Caspase3*, and *BAD*; the mitochondrial-related genes *SIRT1*, *Akt2*, and *Polg2*; and the oocyte maturation-related genes *BMP15*, *PFKP*, *GPX1*, *GSS*, *Gas6*, and *Gdf9*. The differential expression of these genes and the control group is consistent with the staining results in Figure 2. SOD1 and SOD2 are key antioxidant enzymes and play a role in scavenging oxygen free radicals such as superoxide anions in the body. CAT decomposes H_2_O_2_ into oxygen and water [34]. The upregulation of antioxidant enzyme activity by *α*-KG is consistent with the results of decreased ROS. Polg2 is the catalytic subunit of DNA polymerase responsible for mtDNA replication in mitochondria. BIRC5 can prevent apoptosis and plays an important role in maintaining the integrity and function of mitochondria. The upregulation of these genes by *α*-KG protects the function of mitochondria in oocytes, making them more active and more evenly distributed, in order to reach a state of cytoplasmic maturation. We also tested the differential expression of genes related to pluripotency in blastocysts, and Oct4 and Sox2 were significantly upregulated. Antioxidants in IVM have been shown to interact with the expression of genes related to the development of mature oocytes and/or the production of blastocysts.

Excessive oxidative stress is followed by apoptosis. There are two main pathways related to apoptosis, one of which is activation of the caspase cascade, mediated by mitochondria [35]. This pathway is basically controlled by the transmission of cytochrome c and the activation of Caspase9. Mitochondria play an irreplaceable role in this apoptosis pathway. Bcl2 family proteins play an important part in regulating the release of cytochrome c into the cytoplasm. After cytochrome c enters the cytoplasm, it combines with the Caspase9 promoter to activate the gene, to produce apoptotic bodies, which activate Caspase3 and eventually leads to apoptosis [36]. The Bcl2 family mainly includes antiapoptotic proteins, including Bcl2, and proapoptotic proteins, including Bax. Bcl2 can bind to mitochondria and prevent cytochrome c from entering the cytoplasm from mitochondria, thereby exerting an antiapoptotic function [37]. However, the proapoptotic protein Bax can insert into the outer mitochondrial membrane and increase its permeability, resulting in a decrease in *ΔΨ*m and cytochrome c release [38]. Therefore, Bcl2 plays a central role in inducing the collapse of MMP. Our results showed that, compared with the control group, the addition of *α*-KG group significantly inhibited the early apoptosis of porcine oocytes. At the protein level, the expression of Caspase3 was reduced and the expression of Bcl2 was enhanced, and the expression of Caspase3 in the blastocyst was also reduced. These findings indicate that *α*-KG can improve embryonic development by reducing ROS and thus reducing apoptosis in oocytes.

Based on the strong antioxidant capacity of *α*-KG, we turned our attention to the classic oxidation pathway. *α*-KG is the precursor of glutamine, which can increase the level of Nrf2 and promote the binding of Nrf2/AREr3 to the *Bcl2* gene promoter, thereby promoting the expression of the *Bcl2* gene [39]. Researchers have found an antioxidant response element in the reverse chain of the *Bcl2* promoter. ARER3 in the promoter region is the binding site for Nrf2, so Nrf2 can directly upregulate the transcription level of Bcl2, thereby inhibiting the release of Bax and cytochrome c and ultimately inhibiting the occurrence of cell apoptosis [40]. The Keap1-Nrf2/ARE signaling pathway is the most important antioxidant signaling pathway in the body. After being activated, it can trigger the production of multiple downstream target proteins. The expression of these target genes can coordinate the steady state of redox balance and enhance the body's resistance to oxidative stress [41]. The downstream antioxidant proteins and enzymes regulated by Nrf2 combined with ARE are mainly SOD1, SOD2, CAT, HO-1, GPX4, and *γ*-GCS. The expression levels of these genes increased after treatment with *α*-KG. Quantitative results from blastocysts showed that *α*-ketoglutarate upregulated the expression of Nrf2. However, there is no concrete evidence regarding the effect of *α*-KG on the Nrf2 signaling pathway and its subsequent effects on porcine embryonic development and vitality. In this study, we explored the mechanism underlying the effects of *α*-KG and Nrf2 on oocyte maturation. We used brusatol, a specific inhibitor of Nrf2, to treat oocytes, and set up a group cotreated with brusatol and *α*-KG. We found that brusatol had a significant inhibitory effect on Nrf2, and *α*-KG upregulated the level of Nrf2 protein in pig embryos and promotes embryonic development. This finding is consistent with a recent report that activation of the Nrf2 signaling pathway protects oocytes from oxidative stress by enhancing the transcription of antioxidant enzymes [42]. When we added brusatol and *α*-KG to the medium simultaneously, this effect disappeared, possibly because the activity of Nrf2 is inhibited. This result shows that *α*-KG does upregulate the expression of Nrf2, and activates a variety of downstream antioxidant proteins and enzymes to alleviate oxidative stress. The upregulation of Nrf2 promotes the transcription of Bcl2, thereby inhibiting apoptosis and improving cell quality and the embryonic development potential of oocytes. The study demonstrates the importance of *α*-KG on reproductive aging in mammals including mice, pigs, and humans [43], and we think *α*-KG supplementation improves meiotic maturation of porcine oocytes and promotes the development of PA embryos by activating cellular antioxidant response mechanisms.

## 5. Conclusions

**Figure 10:**
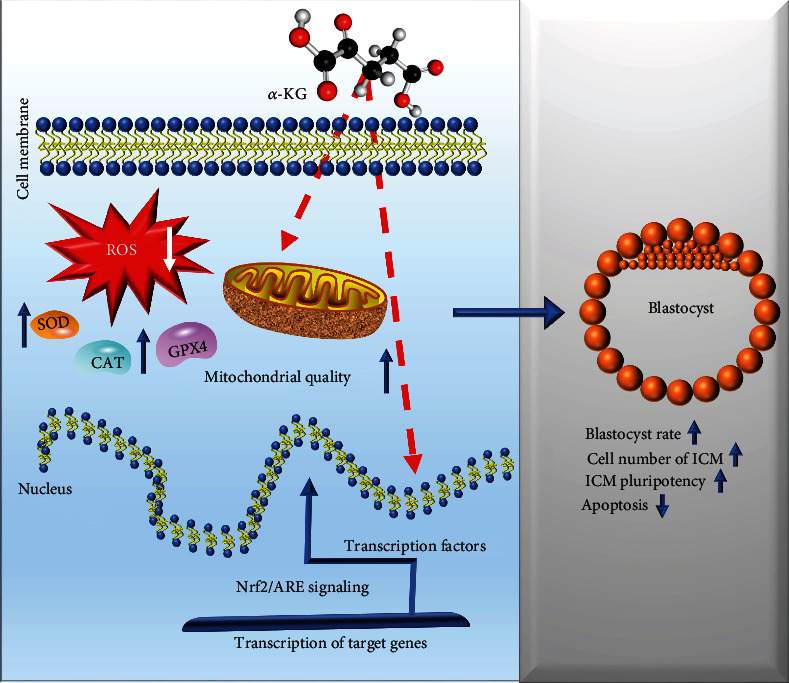
Proposed mechanism of the effect of *α*-KG supplementation during IVM on the developmental competence of pig embryos. *α*-KG can reduce oxidative stress and apoptosis and improve mitochondrial function in oocytes. The high quality and hatching rate of embryos developed from *α*-KG-treated oocytes could be accredited to the regulation of *Nrf2*/*ARE* signaling.

In general, our study showed that adding *α*-KG, an exogenous antioxidant, into *in vitro* maturation systems under oxidative stress conditions can improve embryo quality, reduce the level of early cell apoptosis, and promote embryonic development by increasing GSH content, reducing ROS accumulation, and increasing mitochondrial function. *α*-KG supplementation also activates cellular antioxidant response mechanisms by activating NRF2 as a transcription factor and subsequently its downstream antioxidant genes. These findings will contribute to improving the *in vitro* production of embryos (Figure 10).

## Data Availability

The data that support the findings of this study are available from the corresponding author upon reasonable request.

## Supplementary Materials

Supplementary Materials S-table 1: effects of different doses of *α*-KG addition on porcine IVM oocytes. S-table 2: the primers used in RT-qPCR analysis.
