# Supplementary material for: α-Ketoglutarate Improves Meiotic Maturation of Porcine Oocytes and Promotes the Development of PA Embryos, Potentially by Reducing Oxidative Stress through the Nrf2 Pathway
Source: Oxid Med Cell Longev. 2022 Feb 21;2022:7113793. doi: 10.1155/2022/7113793 (PMC8885182; doi:10.1155/2022/7113793)
Supplement: Supplementary Materials — S-table 1: effects of different doses of α-KG addition on porcine IVM oocytes. S-table 2: the primers used in RT-qPCR analysis. [file 7113793.f1.docx]

S-table1: Effects of different dose of α-KG addition on porcine IVM oocytes

| Groups | Repeats | Oocytes | Maturation rate %（Mean±SEM） |
| --- | --- | --- | --- |
| Con | 4 | 363 | 64.7±1.20^a^ |
| 10 μM | 4 | 370 | 67.89±0.43^a^ |
| 20 μM | 5 | 469 | 76.83±1.05^b^ |
| 50 μM | 4 | 361 | 59.73±2.08^ac^ |
| 100 μM | 4 | 375 | 56.5±0.40^c^ |

Different superscript showed significant differences（*p<0.05*）, S.E.M：standard error of mean.

S-table 2: The primers used in RT-qPCR analysis.

| Gene name | Primer Sequence | Product size |
| --- | --- | --- |
| BMP15 | F：GCTGGAGTTGTACCAGCGTT  R：GCTACCCGGTTTGGTCTCAG | 171 |
| Bax | F：GAGCAGATCATGAAGACAGGGG  R：AATGCGCTTGAGACACTCGC | 150 |
| PFKP | F：TCGAGAGCAACCTGAACACC  R：CACGGCGAACATCTTGTGTC | 179 |
| GPX1 | F：GCTCGGTGTATGCCTTCTCT  R：CTGATGTCCAAACTGGTTGC | 212 |
| GSS | F：CATCCACAAGCAAGTCCTGA  R：TTGGCAGCTTCTTTGGTCTT | 219 |
| Gas6 | F：CTA CTC CTG CCT GTG TGA CG  R：GGA TGT CCT CAC AGG TGC TC | 188 |
| Gdf9 | F：ACTCCAGAGCTTTGCGCTA  R：CCTGATGGAAGGGTTCCTGTC | 169 |
| BCL2 | F：TTCTTTGAGTTCGGTGGGG  R：CCAGGAGAAATCAAATAGAGGC | 195 |
| Sphk1 | F：CTACGAGCAGGTGACGAATG  R：AGGCTGAGCACGGAGAAGAC | 132 |
| BCL-xl | F：GGTACCGGAGGGCATTCAGT  R：TCCCGGAAGAGTTCGTTCAC | 100 |
| BIRC5 | F：ACCACCGCATCTCCACATTT  R：TGGGACAGTGGATGAAACCG | 96 |
| P53 | F：CTTTGAGGTGCGTGTTTGTG  R：TGGGCAGTGCTCGCTTA | 122 |
| Caspase-3 | F：CGTGCTTCTAAGCCATGGTG  R：GTCCCACTGTCCGTCTCAAT | 186 |
| BAD | F：TTTGAGCAGAGTGAGCAGGAA  R：TCTGGGTAAGAGCTGTGGC | 218 |
| SOD2 | F：AGACCTGATTACCTGAAAGC  R：CTTGATGTACTCGGTGTGAG | 110 |
| SOD1 | F：TCCATGTCCATCAGTTTGGA  R：AGTCACATTGGCCCAGGTCTC | 131 |
| CAT | F：GAGCCCAGCCCTGACAAGATGC  R：CCAAGGCCGAATGCGTCTGTT | 229 |
| Gene | Primer Sequences (5’-3’) | Product length (bp) |
| GPX4 | F：ATTCTCAGCCAAGGACATCG  R：CCTCATTGAGAGGCCACATT | 93 |
| SIRT1 | F：TTGATCTTCTCATTGTTATTGGGTC  R：ACTTGGAATTAGTGCTACTGGTCTTA | 62 |
| Akt2 | F：CTGCTGCTGAGGAGATGG  R：GGATGACTTTGCCGAACG | 111 |
| Polg2 | F：GTCATCGACTCCTGTGGTGG  R：AAGTTTGTTTCCCTTCCGGC | 98 |
| IGF2R | F：AAGAGTGCTCTTCCGTAG  R：TGTCATAGCGGAAGAACTTG | 156 |
| Nrf2 | F：GCCCAGTCTTCATTGCTCCT  R：AGCTCCTCCCAAACTTGCTC | 115 |
| Keap1 | F：ACCCAATTTCTGCCCCTGAG  R：ACTTGACCTGCAGCGTAACA | 214 |
| Oct4 | F：AAGCAGTGACTATTCGCAAC  R：CAGGGTGGTGAAGTGAGG | 136 |
| Sox2 | F：GCAACCAGAAGAACAGCCCAGA  R：GTTGTGCATCTTGGGGTTCTCTTG | 110 |
| Nanog | F：CTTCACCAATGCCTGAGGTTTATG  R：AGGGCTGTCCTGAATAAGCAGATC | 134 |
